# Exosome-derived microRNAs: emerging players in vitiligo

**DOI:** 10.3389/fimmu.2024.1419660

**Published:** 2024-07-08

**Authors:** Wenquan li, Yaobin Pang, Qingying He, Zongzou Song, Xin Xie, Jinhao Zeng, Jing Guo

**Affiliations:** ^1^ School of Clinical Medicine, Chengdu University of Traditional Chinese Medicine, Chengdu, China; ^2^ Dermatological Department, Hospital of Chengdu University of Traditional Chinese Medicine, Chengdu, China

**Keywords:** exosome, microRNAs, vitiligo, autoimmunity, biomacromolecule, oxidative stress

## Abstract

Exosome-derived microRNAs (miRNAs) are biomacromolecules and nanoscale extracellular vesicles originating from intracellular compartments that are secreted by most cells into the extracellular space. This review examines the formation and function of exosomal miRNAs in biological information transfer, explores the pathogenesis of vitiligo, and highlights the relationship between exosomal miRNAs and vitiligo. The aim is to deepen the understanding of how exosomal miRNAs influence immune imbalance, oxidative stress damage, melanocyte-keratinocyte interactions, and melanogenesis disorders in the development of vitiligo. This enhanced understanding may contribute to the development of potential diagnostic and therapeutic options for vitiligo.

## 1 Introduction

Vitiligo is recognized as an autoimmune disorder characterized by the progressive destruction of epidermal melanocytes (1, 2). It occurs due to a dynamic interaction between genetic and environmental factors, leading to autoimmune destruction of melanocytes. Defects in melanocyte adhesion and increased oxidative stress further augment the immune response in vitiligo (3).

Exosomes are extracellular vesicles found in various biofluids and tissues, ranging in diameter from 40 to 160 nm (averaging 100 nm).They are released by all cells as part of normal physiology and in response to abnormalities (4). Exosomes deliver bioactive cargoes, such as proteins, mRNAs, miRNAs, and lipids, to recipient cells (5–7). Exosomes are involved in regulating various physiological and pathological processes, such as immune regulation, cell growth, and differentiation (8, 9). Increasing research indicates that exosomes play a significant role in vitiligo (10).

MicroRNAs (miRNAs) are small non-coding RNA molecules (about 22 54 nucleotides) that are essential in regulating various biological processes (11, 12). Cells can selectively package miRNAs into exosomes, which are then secreted to nearby or distant targets (13). Evidence suggests that changes in the profiles of miRNAs delivered by exosomes are closely linked to the progression of vitiligo (10, 14). This feature renders miRNAs as highly interesting therapeutic tools to restore cell functions altered as part of a vitiligo phenotype (15). The present review aims to provide a deep insight into the relationship between exosome-delivered mRNAs and vitiligo and highlight the role of exosomes in immune regulation, oxidative stress, melanocyte-keratinocyte interactions, and melanogenesis. Further understanding of the role of exosomes in vitiligo may contribute to the development of potential diagnostic markers and therapeutic options for vitiligo.

## 2 Exosome-derived miRNAs

### 2.1 Biological functions of exosome

Exosomes are extracellular vesicles characterized by vesicle-like structures with a diameter of 30 ~ 150 nm, wrapped by a phospholipid bilayer (10, 16, 17). They are present in almost all types of prokaryotic and eukaryotic cells (18). Initially recognized in parasitic cells as debris and “cellular dust” in human platelets, they were believed to have no significant role in biology (19). Later, their role as carriers of bioactive substances was discovered (20). Previously, intercellular communication was thought to be mediated through soluble substances, including cytokines and interleukins. However, an increasing number of studies now suggest that exosomes are the primary channel for long-distance communication between various cell types (4, 21). Exosomes are thought to originate from multivesicular bodies (MVBs),which form intraluminal vesicles through dual invaginations of the plasma membrane (22). The plasma membrane primarily invaginates to form disc-shaped structures called early sorting endosomes, which contain phagocytic proteins and genetic material from the cytoplasm as well as cytosolic proteins produced by membrane invagination. Early sorting endosomes then mature into late sorting endosomes, which eventually form MVBs through secondary fusion of endosome-restricted membranes. The resulting MVBs are either degraded by autophagosomes or lysosomes, or are released as exosomes into the extracellular space after fusing with the plasma membrane (18, 23).

Exosomes have been reported to carry signaling molecules such as proteins, DNA, RNA, enzymes, and even organelles (24–27). They are present in a variety of biological fluids, including blood, urine, saliva, and breast milk (27, 28). Exosomes contain two types of proteins: conserved proteins and exosome-specific proteins (10). Conserved proteins are similar proteins that appear in each exosome and include cluster of differentiation (CD) 36, CD81, CD9, CD82 (29, 30). Heat shock protein (Hsp) 500, Hsp70, Hsp90, ALIX, and tumor susceptibility gene 101 (TSG101) (31–33),which serve as pan-markers for the common detection of exosomes (34). The composition of exosome-specific proteins depends on the cellular origin of the exosome and may change depending on the physiological changes and stimuli acting on the cell; for example, exosomes derived from mesenchymal stem cells or stromal cells (MSC) are endowed with immunosuppressive and tumor-homing capabilities (18).

Notably, exosomes are not only involved in a variety of physiological processes such as inflammatory responses (35, 36)、skin trauma repair (37)、angiogenesis (38)、immune responses (39, 40) and immune surveillance (41), but also play an important role in the pathological state of several diseases. For example, exosomes are believed to contribute to skin immunity and melanogenesis, thereby supporting the maintenance of skin homeostasis (41, 42). In addition, they enhance the function of regulatory T cells and suppress the activity of CD8+ cells and natural killer (NK) cells (43, 44). They are closely associated with the pathogenesis of other diseases as well, such as autoimmune diseases (45) and skin diseases (4, 46). Also, the contents carried by exosomes reflect the physiological or pathological condition of their cellular origin. Many studies have reported that the RNA profile of exosomes of disease origin or pathological cells differs from that of exosomes of healthy cellular origin (47–49). Exosomal RNA has been used or has the potential to be used in the diagnosis and treatment of multiple diseases (50, 51). For example, a multicenter cohort study showed that a small RNA was specifically enriched in salivary exosomes, tissues and cells of patients with pancreatic ductal adenocarcinoma, and detection of a double signal composed of these small RNAs was able to diagnose patients with pancreatic ductal adenocarcinoma with high sensitivity (90.50%) and specificity (94.20%) (52). In melanoma patients, the levels of circulating exosomal PD-L1 are positively correlated with the levels of IFN-γ, and exosomal PD-L1 is also a marker of immune activation after the initiation of treatment with PD-1-blocking antibodies, (53, 54). The analysis of exosomes contributes to a better understanding of the biological mechanisms of disease and helps to develop new diagnostic and therapeutic approaches. **Table 1** summarizes the role of exosomes in autoimmune diseases.

**Table 1:** The role of exosomes in autoimmune diseases.

Functions	Mechanism	Impact on Vitiligo	Ref
Regulation of Immune Response	Elicit adaptive and innate immune reactions, delivering DNA, manipulating gene expression via miRNA.	Presenting antigens and manipulating immune cell activity	(4, 55)
Antigen Presentation	Directly present peptide antigens to specific T cell	present melanocyte antigens to immune cells	(4, 56)
Innate Immune Response	Exosomes can limit complement-mediated lysis, trigger myeloid-derived suppressor cell activation, and induce an immunosuppressive macrophage phenotype.	Influence innate immune responses	(57, 58)
Oxidative stress	The delivery of antioxidant enzymes, induction of antioxidant response, regulation of cellular redox balance	Related to the dysfunction of melanocytes	(59–61)
Immunoregulatory Molecules	Exosomes can present PD-L1, suppressing T cell function. Mast cell-derived exosomes can induce B and T cell proliferation.	Potentially affecting immune evasion and response to melanocytes	(61–63)

### 2.2 MiRNAs

MiRNAs are 18-25 nucleotide non-coding RNAs generated by nearly all cells in the body and primarily transcribed by RNA polymerase (64). They are initially transcribed into large precursors called primary miRNAs (pri-miRNAs), which are then transcribed in the nucleus by RNA polymerase II or RNA polymerase III and cleaved into pre-miRNAs by Drosha and its cofactors (65). These pre-miRNAs are exported to the cytoplasm and processed by Dicer to produce mature miRNA products (66, 67). MiRNAs have been shown to play various roles in numerous diseases and physiological states, both *in vitro* and *in vivo*, through their functional transfer via exosomes. They primarily regulate gene expression by recognizing homologous sequences and interfering with transcriptional, translational, or epigenetic processes (68). MiRNAs are highly conserved during evolution and participate in essential biological processes such as cell proliferation, differentiation, metabolism, apoptosis, development, and aging processes (66). Over 2600 mature human miRNAs have been reported to date (69) and are expressed in distinct spatial and temporal patterns during embryonic and postnatal development, as well as in adult tissues (70).

### 2.3 Exosome-derived miRNAs

Exosomes offer a unique mode of intercellular communication, where miRNAs produced and released by one cell are taken up by distant cells, affecting gene expression in health and disease (71–73). Exosome-derived miRNAs have several advantages over intracellular and cell-free blood sources of miRNA (74). For instance, exosomes can protect miRNAs from degradation *in vivo* and enable superior systemic retention compared to liposomes, allowing miRNAs to function at distant sites (75). Moreover, the miRNA profiles derived from exosomes differ significantly from those originating from their cells of origin, indicating that numerous miRNA species are selectively encapsulated into exosomes (76).

Currently, various attempts have been made to elucidate the mechanisms of loading and sorting miRNA into exosomes. Several pathways for loading miRNAs into exosomes have been described. The first pathway originates from the inherent structure of miRNA with a 3′ end adenylation and uridylation, which is essential for recognition by AGO2.miRNAs with an adenylated 3′ end are predominantly found in cells, while miRNAs with a uridylated 3′ end are sorted into exosomes, as demonstrated in RNA sequencing research on human B cells and their associated exosomes (77). The second sorting pathway involves sphingomyelinase 2-dependency, which is the first molecule identified to be associated with miRNA loading into exosomes. Overexpression of sphingomyelinase 2 promotes miRNA sorting into exosomes, while its inhibition produces the opposite result (78). The third sorting pathway involves the miRNA-induced silencing complex (miRISC) association. The miRISC mainly consists of miRNA, miRNA-repressible mRNA, GW182, and AGO2. The RNA-binding protein mediates the final sorting pathway. SYNCRIP selectively sorts miRNAs and has a 4-nucleotide motif near the 3′ end, independently of hnRNPA2B1 (79). The fourth pathway is the sumoylated heterogeneous nuclear ribonucleoprotein (hnRNP; primarily including hnRNPA2B1, hnRNPA1, and hnRNPC)-dependent pathway, which binds to miRNAs and facilitates their loading into exosomes (80). Y-box protein 1 can selectively sort miR-223 into exosomes in HEK-293T cells (81). Additionally, a growing number of studies clarify the mechanisms by which miRNA sorting occurs in exosomes or cells. Accumulating research evidence demonstrates that miRNAs possess sorting sequences that determine their exosomal secretion or cellular retention, and different cell types, including endothelium, brown and white adipocytes, muscle, and liver, preferentially use specific sorting sequences, thus determining the exosomal miRNA profile of that cell type. Insertion or knockdown of these CELLmotifs or EXOmotifs into a miRNA alters their retention in the cells that produce or secrete them into exosomes. Two RNA-binding proteins, Fus and Alyref, are involved in the export of miRNAs carrying one of the strongest EXOmotifs, CGGGAG. Increased miRNA delivery mediated by EXOmotifs leads to enhanced inhibition of receptor genes in distant cells (82, 83).

**Figure 1:**
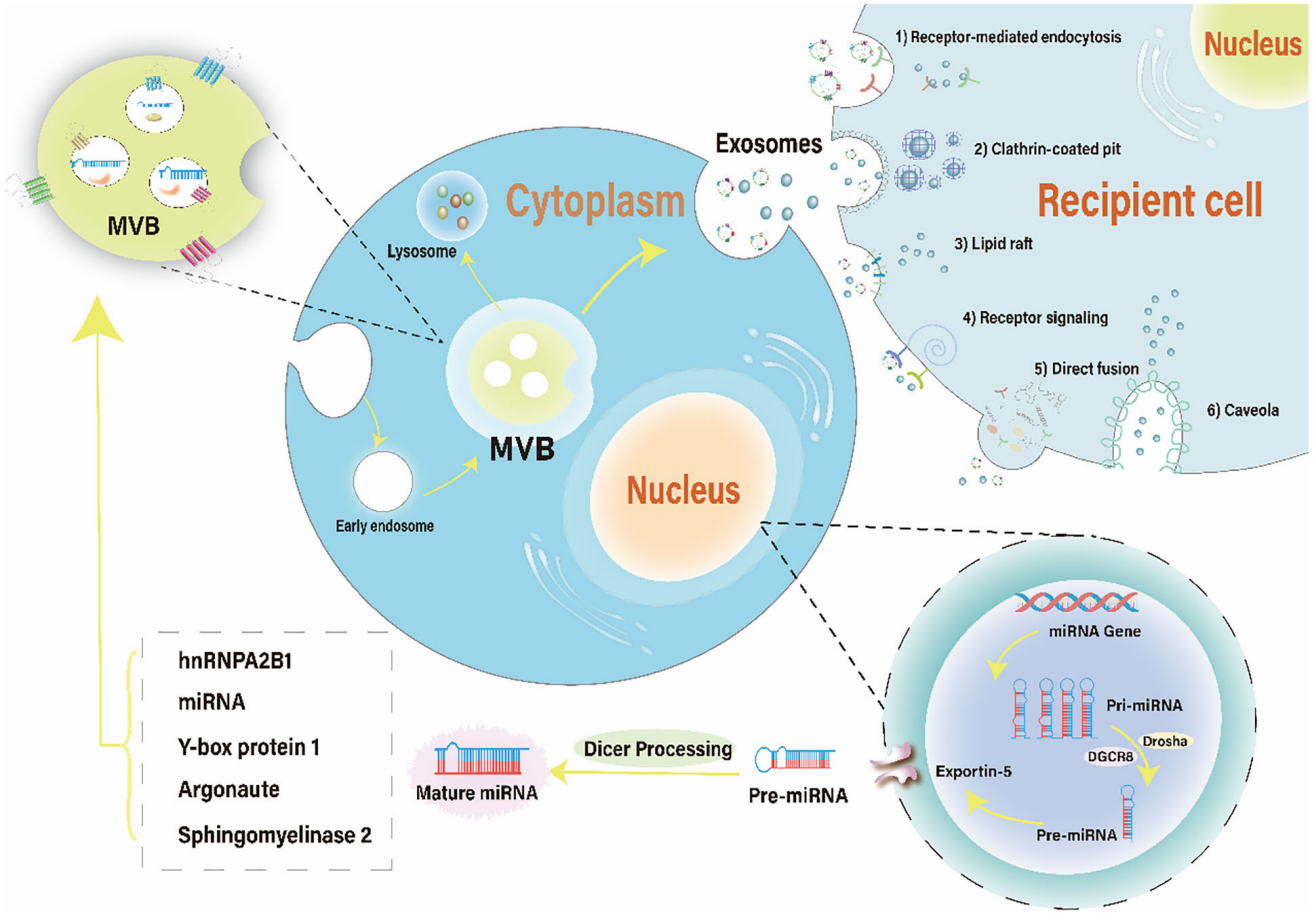
The brief mechanism of exosomal miRNA and uptake by recipient cells. The biogenesis of miRNA involves transcription of a pri-miRNA, formation of pre-miRNA, translocation to the cytoplasm, and maturation of the miRNA. miRNAs containing different RNA motifs can be loaded into MVBs via various RNA-binding proteins. MVBs can either follow a degradation pathway by fusing with lysosomes or release the intraluminal vesicles as exosomes into the extracellular space. Recipient cells can uptake exosomal miRNAs through six pathways: receptor-mediated endocytosis, clathrin-coated pit, lipid raft, receptor signaling, direct fusion, and caveola. MVBs, multivesicular bodies.

Following intracellular loading of miRNA into exosomes, binding to their target cells’ membrane regions, and through plasma membrane fusion, miRNA-containing exosomes are eventually released. Six possible methods of exosome miRNA uptake have been documented, and are illustrated in **Figure 1**: 1) Receptor-Mediated Endocytosis. Mechanisms involving clathrin- or caveolae-regulated endocytosis, phagocytosis, or macropinocytosis (84). 2) Clathrin-Coated Pits (CCPs).CCPs form as a result of the AP2 complex recognizing membrane and cargo, subsequently recruiting clathrin triskelia (85, 86). 3) Lipid Rafts. The role of lipid rafts in the intracellular mechanism of exosome entry has been investigated. Studies show that disrupting dendritic cell lipid rafts significantly reduces and blocks exosome uptake, indicating that lipid rafts are involved in this process. However, the precise mechanism of this pathway remains to be elucidated (85). 4) Receptor signaling. MiRNAs docked in exosomes can be assimilated by recipient cells by directly targeting corresponding cell membrane receptors, generating intracellular signaling, and subsequently activating or inhibiting related pathways such as the Ca2+/MEK/ERK signaling pathway (87, 88). 5) Direct Fusion. Targeting the recipient cells’ surface immediately and fusing with the cell membrane (89). 6) Caveolae. Exosomes have been shown to follow a different endocytic pathway than liposomes through caveolae internalization (90). Co-localization studies between fluorescently labeled extracellular vesicles (EVs) and cholera toxin B exhibit poor co-localization in recipient cells, suggesting that EVs utilize independent caveolin (91).

## 3 Pathogenesis of vitiligo

Vitiligo is an autoimmune disease (92), and its exact pathogenesis is not fully understood. Two main biological process have been reported to explain its pathogenesis.

### 3.1 Autoimmune

The immune system attacks and destroys melanocytes, leading to the development of white patches. A cross-sectional study by Gill L et al. showed that about 20% of vitiligo patients have at least one autoimmune disease in combination throughout the disease progression, suggesting that vitiligo’s occurrence is likely related to immune mechanisms (93). Immunosuppressive agents have shown better results in vitiligo treatment, further suggesting that autoimmunity can influence vitiligo development (94, 95). The immunological mechanism of vitiligo development is closely related to humoral and cellular immunity, especially cellular immunity, where many cytokines have been found to play a direct or indirect regulatory role. In cellular immunology, the main influences on vitiligo development are helper T cells (Th), cytotoxic T cells (CTL), and regulatory T cells (Treg). In the whole process of vitiligo disease, cellular immune function regulation is mainly acted upon by Th1 and Th17 cells, and interleukin-17A (IL-17A) is significantly increased in the cellular expression level of patients’ serum when Th1 and Th17 cells’ levels significantly increase in the peripheral blood (96).

CD8 cytotoxic T cells occupy a crucial position among the whole T lymphocytes and are capable of toxic killing of cells. These cells can specifically target and destroy melanocytes. As their numbers increase, their ability to kill melanocytes intensifies, leading to a greater extent of skin lesions in patients (97, 98). Activated CD8+ T cells secrete various cytokines, such as IFN-γ and TNF-α. These cytokines have multiple effects, including inducing melanocyte apoptosis, inhibiting melanocyte proliferation, and reducing pigment production (99).

Recent studies increasingly suggest that dysfunction of Treg cells significantly contributes to the onset of vitiligo (100, 101). In the lesional skin of vitiligo patients, a reduction in the number of Tregs is often observed. Similarly, flow cytometry analysis of circulating Tregs shows that the number of Tregs in the blood of vitiligo patients is reduced compared to healthy individuals (102–105). Treg cells are a subpopulation of T cells with tolerance to self-antigens, and they help prevent autoimmune diseases. When the number of Treg cells decreases or their function is impaired, they fail to inhibit the proliferation of CD8 and CD4 T cells. The increase in CD8 T cells may destroy melanocytes via the granzyme B or perforin pathways, or by releasing cytokines that promote melanocyte destruction. This ultimately leads to the loss of melanocytes through apoptosis (101, 102). Currently, studies based on single-cell RNA sequencing of human vitiligo patients indicate that CCL5-CCR5 cytokine signaling acts as a connecting axis between effector CD8 T cells and Treg cells (106).

Additionally, the differential ability of fibroblasts in various parts of the patient to respond to gamma interferon determines not only their capacity to recruit CD8+ T cells but also the preferred locations for vitiligo onset. This recruitment mechanism in vitiligo pathogenesis coordinates the accumulation of CD8+ T cells at damaged skin junctions. As a result, these cells can continuously attack melanocytes in healthy areas, causing a progressive expansion of depigmented regions (107). Secretion of interferon-γ (IFN-γ) by natural killer (NK) cells and innate lymphocytes (ILC) induces the expression of C-X-C motif chemokine receptor (CXCR) 3B on the melanocyte surface and the release of C-X-C motif chemokine ligand 9 (CXCL9), C-X-C motif chemokine ligand 10 from keratinocytes and melanocytes (CXCL10), and C-X-C motif chemokine ligand 11 (CXCL11). CXCL10 then activates CXCR3B and triggers apoptosis of melanocytes (108).

### 3.2 Oxidative stress

Multiple factors and mechanisms have been proposed for the etiopathogenesis of vitiligo, among which oxidative stress has been widely accepted as a key factor in initiating melanocyte loss. Oxidative stress is a reaction process caused by the accumulation of free radicals induced by an altered environment, including inflammation and mitochondrial dysfunction (109). Excessive reactive oxygen species (ROS) have been identified as essential free radicals that exacerbate oxidative stress and aggravate tissue dysfunction (110). However, when ROS production severely exceeds clearance capacity, ROS can accumulate and affect cells, leading to DNA damage, protein misfolding and chromosome instability (111). This theory suggests that increased oxidative stress caused by factors such as sun exposure, hormonal imbalance, or certain chemicals damages melanocytes and leads to vitiligo. The imbalance in redox status caused by oxidative stress, including excessive production of ROS and reduced activity of the antioxidant system in the skin, reduce the resistance of melanocytes to exogenous or endogenous stimuli, ultimately impairing normal defense mechanisms and leading to melanocyte deficiency (112). Low levels of catalase (CAT), glutathione peroxidase (GPx), glucose-6-phosphate dehydrogenase (G6PD), and superoxide dismutase (SOD) have been demonstrated in the epidermis and blood of vitiligo patients (113). These enzymes are closely associated with a state of chronic oxidative stress (OS), which may lead to melanocytes being programmed to become “senescent melanocytes.” They recruit immune cells to kill senescent cells by expressing various cytokines (114). In addition, excessive accumulation of reactive oxygen species (ROS) induces the production of damage-associated molecular patterns (DAMPs) and the release of melanosomal antigens that activate innate immunity (98).

There is mounting evidence revealing an association between oxidative stress and autoimmunity (115–117). ROS can activate immune macrophages and dendritic cells, leading to these cells releasing more cytokines (such as TNF-α, IL-6, IL-1β), thereby affecting melanogenesis (114, 118). Autoimmune responses can further exacerbate oxidative stress. For example, when T cells attack melanocytes, they produce more ROS, worsening the local oxidative stress environment (119).

**Figure 2:**
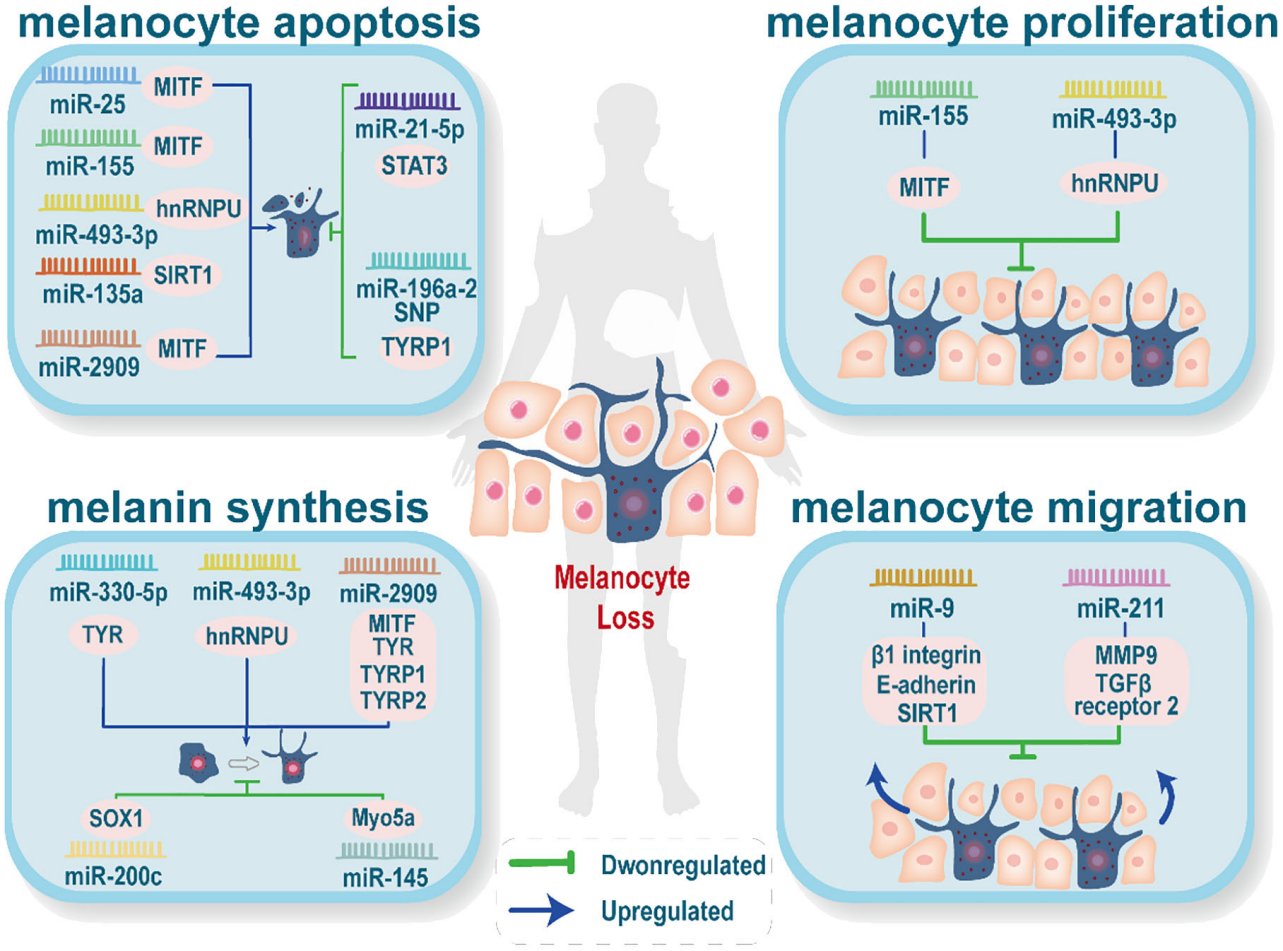
Abnormally expressed miRNAs affect melanin production. Abnormal expression of miRNAs reduces skin melanin through four processes: melanocyte apoptosis, melanocyte proliferation, melanin synthesis, and melanocyte migration. Each process may be influenced by several abnormally expressed miRNAs, and the same abnormally expressed miRNA may be involved in more than one process.

Additionally, MiRNA plays a crucial role in immune regulation and oxidative stress in vitiligo, contributing to the process of melanocyte death (14). For example, the increased expression of miR-25 promotes melanocyte dysfunction and oxidative stress-induced damage (120). MiR-211 inhibits UVB-induced human melanocyte migration by suppressing MMP9 (matrix metalloproteinase 9) and p53 (121). **Figure 2** depicts the abnormally expressed miRNAs involved in melanocyte destruction (adapted from reference (14)).

## 4 Roles of exosomal miRNAs in vitiligo

The vital role of miRNAs encapsulated in exosomes in vitiligo is increasingly recognized. High-throughput sequencing of clinical samples has shown that the exosomal miRNA expression profiles differ between patients with vitiligo and healthy individuals, with increased expression of miR-493-3p, miR-370-3p, and miR-143-5p, and decreased expression of miR-885-5p, miR-16-5p, miR-92a-3p, and miR-92b-3p (96). In addition, exosome MiR-200c released from keratinocytes in vitiligo patients was significantly downregulated (122). However, the expression of Exosomal MiR-493-3p was significantly increased in the blood and surrounding tissues of vitiligo patients (123). **Table 2** summarizes the reported exosomal miRNAs that may be associated with vitiligo pathogenesis and their potential mechanisms of action.

**Table 2:** Exosomal miRNAs in vitiligo.

miRNA	Sample	Target and expression	Observation/impact of miRNA	Ref
miR-2478	Milk Exosome-Derived	Rap1a↓	Inhibition of melanogenesis through the Akt-GSK3β pathway	(124)
miR-200c	Normal human epidermal keratinocytes	↓	Regulate the activation of β-catenin in melanocytes by mediating SOX1	(122)
miR-330-5p	Keratinocytes	↑	Downregulating TYR in melanocytes inhibits melanin pigmentation of melanocytes	(125)
miR-675	Normal human skin keratinocytes	MITF↑	It inhibits the level of stem cell factor (SCF) and basic fibroblast growth factor (bFGF) in keratinocytes	(126, 127)
miR-493-3p	Serum in the coculture system	↑	Resulted in a significant increase in ROS and melanocyte apoptosis, as well as a decrease in melanocyte proliferation and melanin synthesis	(123, 128)
miR-375-3p	Plasma	X-linked inhibitor of apoptosis protein↓	Induced keratinocyte cell death	(129)
miR-3196	Caucasian keratinocytes	MITF-M↑ Rab27a↓	Regulation of melanin synthesis through TYR	(130)
miR-181a-5p	Human amniotic stem cells	MITF	Inhibition of downstream melanogenic genes (tyrosinase, TRP1, and TRP2)	(131)
miR-199a	Human amniotic stem cells	mTOR↓	Degradation of melanosome	(131)
miR-21-5p	Peripheral Blood of Vitiligo Patients	Targeting special AT-rich sequence binding protein-1	Inhibited melanocytes melanogenesis	(132)
miR-487b-3p	Serum of vitiligo patients	↓	It may accelerate the catabolism of melanocytes and cause their damage in progressive vitiligo	(133)
miR-143-5p	Serum of vitiligo patients/Mouse melanocytes	MITF↑	Melanocytes upon blocking miR-143	(123, 134)
miR-211	Serum of vitiligo patients/keratinocytes	Sirtuin1↑	Protecting vitiligo epidermis from UV-mediated DNA damage	(125, 135)

*↓down-expression, ↑over-expression.

Exosomal miRNAs play multiple roles in the pathogenesis of vitiligo, influencing the development and progression of the disease through various mechanisms such as regulating immune responses, participating in oxidative stress, and affecting melanogenesis. The following will detail the roles of exosomal miRNAs in the pathogenesis of vitiligo.

### 4.1 Immune imbalance

The immunomodulatory function of exosomal miRNAs has been widely reported (136, 137). Exosomes of immune cell origin (e.g., T cells, B cells, macrophages, etc.) have been shown to induce suppressive or active immune responses and to participate in immune regulation and immune stimulation (138). Studies have shown that antigen-presenting exosomes secreted by cells such as dendritic cells and T lymphocytes are enriched with co-stimulatory molecules, antigenic peptide-MHC class I, and antigenic peptide class II molecular complexes, which are presented to the corresponding T cells to activate the immune response (139). Researchers have also found that exosomes from normal immune, non-immune, or pathological cell types can influence immune homeostasis. For example, exosomes from dendritic cells express stimulatory molecules of MHC class I and class II T cells in tumor peptide culture, which subsequently activate CD8+ cytotoxic cells and produce cytotoxicity against mouse tumor cells (140). In addition, exosomes may induce the differentiation of type 1 T helper cells into type 2 T helper cells and reduce T cell differentiation into type 2 T helper cells. They also reduce T cell differentiation to interleukin 17-producing effector T cells (Th17 cells) and increase the number of Treg cells and the level of cytotoxic T lymphocyte-associated proteins (141). Thus, exosomal miRNAs, as intercellular mediators, are likely to be involved in the regulation of immune homeostasis.

Vitiligo and immune imbalance are closely related. The immune imbalance in vitiligo involves two main aspects of the adaptive immune response:

1) Role of CD8+ T cells. Activated CD8+ cytotoxic T lymphocytes (CTL) exhibit a strong skin-homing ability and cytotoxic effect; they kill melanocytes by secreting granzyme B and perforin, leading to skin pigmentation deficiency (142). Current studies have found that IFN-γ reduces mRNA levels of TYR, tyrosinase-related protein 1 (TRP1), and microphthalmia-associated transcription factor (MITF), inhibits melanocyte production, and reduces their number around skin lesions. IFN-γ is mainly derived from NK cells, CD4+ T lymphocytes, and activated CD8+ CTL; however, the main source of IFN-γ in vitiligo patients is CD8+ CTL (143, 144). In conclusion, CD8+ T cells not only kill melanocytes directly but also inhibit melanocyte function by secreting IFN-γ.
2) Imbalance between effector T cells. An imbalance occurs between Treg cells (reduced) and CD8+ CTL (significantly elevated) around the skin lesions of vitiligo patients. This imbalance leads to massive secretion of IFN-γ to kill melanocytes (145, 146). Notably, this imbalance is associated with the secretion of pro-inflammatory factors IL-17, IL-6, and TNF-α by differentiated Th17 cells from CD4+ T cells. These cytokines actively regulate inflammatory and autoimmune responses *in vivo*. Among them, IL-6 acts as a hub between Treg and Th17 cells (147, 148). Currently, miRNA regulation of transcription factor expression, thereby affecting the Treg/Th17 balance, is the most extensively studied aspect of the Treg/Teff balance (149). For example, miR-155 and miR-221-5p target SOCS1, inhibiting the activation of STAT5 (150, 151). SOCS3, a negative regulator of STAT3 activation, is a target of miR-384 and miR-206 (152, 153). In addition to Treg/Th17, the Treg/Th1 and Treg/Th2 balance can also be regulated by various miRNAs. For instance, miR-23a-3p and miR-155 disrupt the Th1/Treg balance by targeting Sirt17 (154, 155).

Exosomes are necessary for the regulation of CD8+ T, and Th17 cells (10). The immunosuppressive effect of exosomal miRNAs on CD8+ T cells has been frequently described in tumor lesions. Exosomes from malignant or immune cells can be taken up by tumor-infiltrating CD8+ T cells and modulate the tumor microenvironment to produce anti-tumor effects (156). Dokyung Jung generated interleukin-2-tethered sEVs (IL2-sEVs) from engineered Jurkat T cells expressing IL2 at the plasma membrane via a flexible linker to induce an autocrine effect. IL2-sEVs increased the anti-cancer ability of CD8+ T cells without affecting Treg and down-regulated cellular and exosomal PD-L1 expression in melanoma cells, causing their increased sensitivity to CD8+ T cell-mediated cytotoxicity. Its effect on CD8+ T and melanoma cells was mediated by several IL2-sEV-resident miRNAs, whose expressions were upregulated by the autocrine effects of IL2. Among the miRNAs, miR-181a-3p and miR-223-3p notably reduced the PD-L1 protein levels in melanoma cells. Interestingly, miR-181a-3p increased the activity of CD8+ T cells while suppressing Treg cell activity (137). In addition, exosomal miRNAs can also induce suppressors of CD8+ T cell activity; tumor-derived exosomes lead to a significant loss of CD27/CD28 and thus induce suppressors of CD8+ T cells (74).

Moreover, exosomes have an important role in the regulation of Treg cells. For example, human umbilical cord MSC-derived exosomes increased the proportion of CD4+CD25+Foxp3+ Treg cells and decreased the proportion of CD4+IL17A+ T cells (Th17 cells) (10). A study showed that MSC-derived exosomes have the capacity to decrease Th17/Treg imbalance in aplastic anemia through sphingosine kinase isoenzyme 1 (SphK1)-mediated exosomal sphingosine 1-phosphate enrichment (S1P) in both *in vivo* and *in vitro* experiments (157). In addition, bone marrow-derived mesenchymal stem cell (BMSC)-derived exosome miR-181a is essential for the maintenance of immune tolerance. It induced CD3+CD1+FOXP3+ Treg cells production via SIRT4/acetylation/FOXP3 (158). BMSC-derived exosomes can partially transfer miR-17a-6p to suppress IL-23 expression and thereby balance the Th17/Treg ratio in aplastic anemia (159).

Exosomes secreted by multiple immune cells can act on different types of immune cells and alter their functions (160). First, the unidirectional transfer of T cell exosomal miRNAs to antigen-presenting cells (APCs) plays a genetic regulatory role during immune synapse formation (161). In addition, it has been reported that Treg cells transfer miRNAs to various immune cells, including helper T cell 1 (Th1) cells, via exosomes to inhibit Th1 cell proliferation and cytokine secretion. The mechanism lies in the fact that miRNA-containing exosomes mediate the silencing of Th1 cell genes by Treg cells through non-cell-autonomous gene regulation. Exosomal miRNAs may cooperate with other Treg cell mechanisms to achieve optimal inhibition (162). This may be one of the reasons for the ability of Treg cells to regulate immunity. B cells release more exosomes upon stimulation with CD40 and IL-4 antigens, and these exosomes initiate dendritic cells (DCs) through transfer, which together with CD4+ T cells activate CD8+ T cells to trigger the killing response of CD8+ T cells (139). Immunosuppressive effects of B-cell-derived exosomes have also been reported, inducing apoptosis of CD4+ T cells (163). Classically activated macrophages secrete exosomes that deliver inflammatory signals to immature macrophages and DCs, stimulating cellular inflammation and releasing more Th1 cytokines (164).

Natural killer (NK) cells secrete exosomal miRNAs that also regulate T cell immune responses. miR-10b-5p, miR-92a-3p, and miR-155-5p, found in NK cell exosomes, specifically target molecules involved in the Th1 response, promote CD4+ T cell activation, and produce the cytokines IFN-γ and IL-2 (165). Increasing evidence suggests that NK cell-derived exosomes can induce IFN-γ secretion and increased T-bet expression, with exosomal miRNAs associated with this effect including miR-20a-5p, miR-25-3p, miR-10b-5p, miR-92a-3p, and miR-155. In addition, NK cell-derived exosomes can drive CD4+ T cell activation and may enhance antigen presentation and co-stimulatory abilities by increasing monocyte polarization toward moDCs. They also indirectly affect T cell responses by upregulating MHC-II and CD86 expression (165). Therefore, NK cell-derived exosomes may influence vitiligo development by affecting IFN-γ secretion and CD4+ T cell activation.

### 4.2 The role of exosomal miRNAs derived from keratinocytes

Skin color is mainly determined by the content and composition of melanin in the epidermis, and the development of vitiligo is closely related to impaired melanin production and transport. Summarizing the available research results, keratinocytes have two roles in skin tone formation. On the one hand, they are melanin-receiving cells. Melanin is synthesized within the melanosomes of melanocytes and transported from its perinuclear region of origin to the cell periphery, where it will be further transferred to the adjacent keratinocytes, forming different skin tones (166). Reportedly, the melanosomes in the keratinocytes in the lesions of patients with stable vitiligo are single and large; however, in contrast, the melanosomes in active vitiligo skin are smaller and less dense (167). Currently, melanin transfer patterns from melanocytes to keratinocytes are described in four main categories:1) Cytophagy. where melanosome-containing melanocytes are phagocytosed by adjacent keratinocytes via dendrites or filamentous pseudopods (168). 2) Membrane fusion, where melanosomes are transferred directly through the membrane tunnel formed between the two cells (169). 3) Shedding and swallowing model, where membrane vesicles containing individual or aggregated melanosomes are released from melanocytes and then phagocytosed by keratin-forming cells (169). 4) Cytosolic-endocytosis. The melanosome membrane fuses with the plasma membrane and secretes naked melanin from the melanocytes (170). In addition, the role of vesicles in this mechanism has been demonstrated, and studies have shown that vesicles containing melanosomes exiting melanocytes are phagocytosed by keratinocytes (168, 171). Transport of melanosomes is also regulated by the small GTPase Rab27a (172), and the role of Rab27 in exosomes has also been reported (173). Thus, melanin transport may also be associated with exosomal miRNAs.

On the other hand, keratinocytes not only passively receive melanosomes transferred from melanocytes but also actively regulate melanocyte function. According to reports, extracellular vesicles released by keratinocytes carry miRNAs that target melanocytes and regulate their state by altering gene expression patterns and enzyme activity (125, 174–178). Studies have shown that exosome protein markers, such as TSG101, Alix, and CD63, were detected in the culture supernatant of keratinocytes (125, 130). Moreover, the expression of miRNAs in exosomes was not affected by RNase intervention in the keratinocyte culture supernatant, as they were encapsulated within the phospholipid bilayer of the exosomes (126). The process of exosomes transferring from keratinocytes to melanocytes has been recently confirmed. The work by Lo Cicero et al. utilized CD63-GFP to track the movement of exosomes in keratinocytes. Initially, CD63-positive regions were detected around the nuclei of keratinocytes, which then moved towards the plasma membrane. During co-culture with melanocytes, it was observed that exosome levels in melanocytes significantly increased after 24 hours of co-culture (130). Furthermore, exosomes derived from keratinocytes carrying miR-330-5p resulted in a significant increase in the expression of miR-330-5p in melanocytes, indicating that nucleic acids from keratinocytes are delivered to melanocytes via exosomes (125).

**Figure 3:**
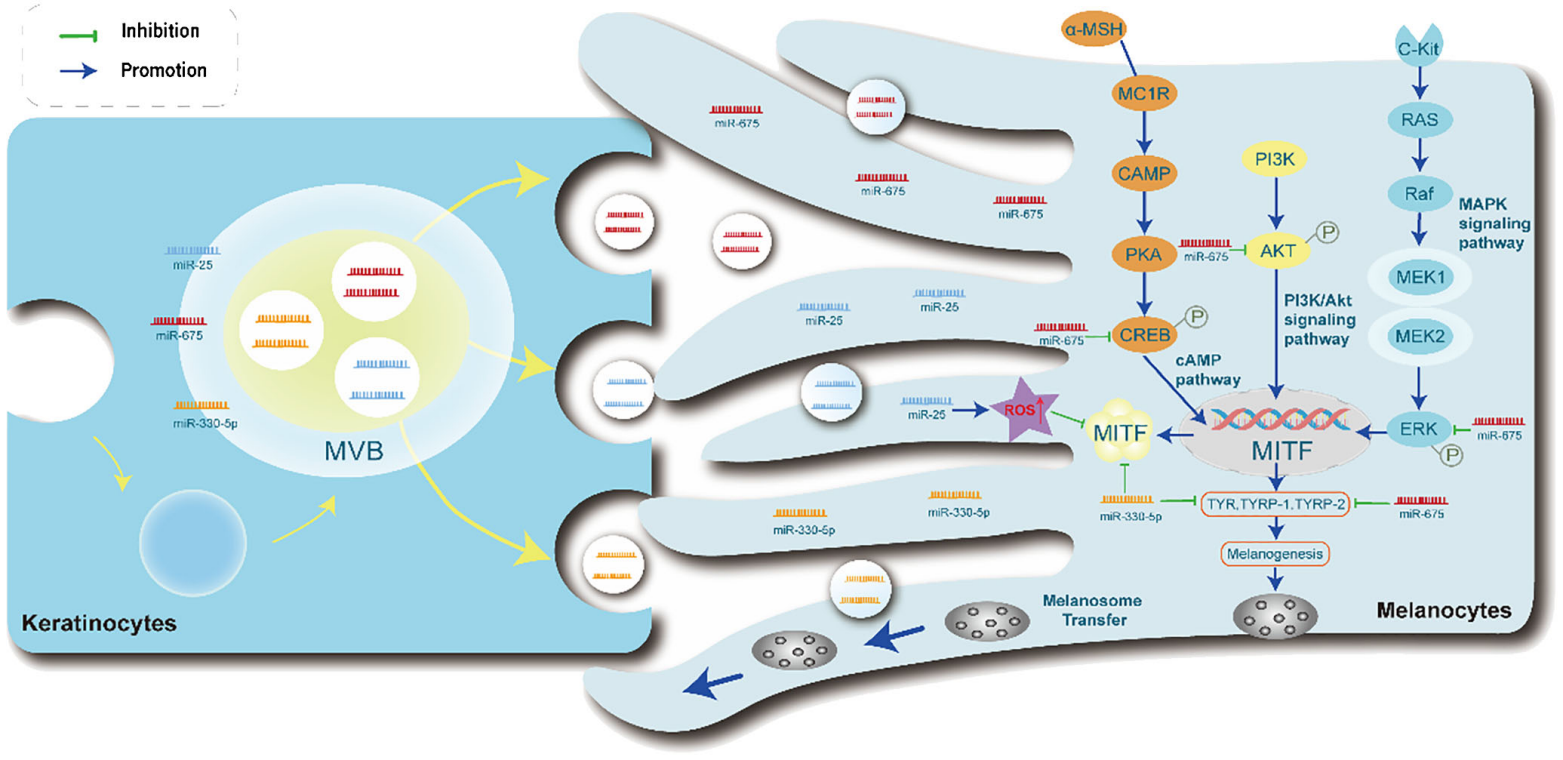
Exosome-derived miRNAs in keratinocytes modulate melanocyte Function. The miRNA-containing exosomes are taken up by melanocytes, followed by the transfer of miRNAs. Furthermore, exosomal miRNAs are involved in the PI3K/Akt, MAPK, and cAMP signaling pathways to regulate the transcription of MITF in melanogenesis. Exosomal miR-675 inhibits the phosphorylation of CREB, ERK, and AKT signaling molecules, subsequently causing the inhibition of TYR, TYRP1, and TYRP2 expression. Exosomal miR-25 promotes ROS increase, suppressing the expression of MITF. Exosomal miR-330-5p directly inhibits the expression of MITF protein, leading to the inhibition of TYR, TYRP1, and TYRP2 expression. PI3K/Akt, phosphatidylinositol 3-kinase/protein kinase B, MAPK, mitogen-activated protein kinase, cAMP, cyclic adenosine monophosphate.

Furthermore, Hai-Xia Shi et al. studies showed that miRNAs were upregulated in EVs from keratinocytes. KEGG pathway analysis indicated that exosome-derived miRNAs can target one or more genes and regulate one or more signaling pathways that affect the biological activity of melanocytes, such as the MAPK signaling pathway, endocytosis, axon guidance, and neurotrophic factor signaling pathways. In addition, PKH67, a green fluorescent dye was used as a marker to study the internalization process of exosomes. The experimental results showed that scattered green fluorescent particles could be seen in melanocytes co-cultured with exosomes derived from keratinocytes. These findings suggest that exosomes secreted by keratinocytes are transported to melanocytes through certain pathways. Exosomes from keratinocytes not only promote the proliferation and dendritic morphology of melanocytes but also enhance their tyrosinase activity and melanin production (175). Another study investigated the effect of UVB radiation on melanogenesis. After irradiation, the number of exosomes released from keratinocytes increased almost twofold. When using keratinocyte exosome modulators D609 and GW4869, the number of melanocytes in the co-culture system was significantly reduced, indicating that the quantitative changes in keratinocyte exosomes are of significant importance in the regulation of human skin color development (179). **Figure 3** summarizes the process of exosome miRNA formation from keratin-forming cells and its uptake by melanocytes, which affects melanin formation.

Unfortunately, there have been limited reports on the study of secreted exosomes from melanocytes to keratinocytes. Current research indicates that UVA radiation is a necessary condition for human melanocytes to secrete exosomes. Under UVA irradiation, the exosome protein markers flotillin-1 and CD63 were detected in the conditioned medium of melanocytes (180, 181). It was also determined that exosomes were released from melanocytes and preferentially absorbed by keratinocytes without cell-to-cell contact, enhancing the proliferation of keratinocytes (182). Petra Wäster purified exosomes from UVA-exposed melanocytes and added them to keratinocytes, which resulted in enhanced anti-apoptotic signal transduction in the keratinocytes (183).

### 4.3 Abnormal melanin production

The influence of exosomes on melanogenesis pathways and their possible role in the pathogenesis of pigment deposition diseases have been proposed, but the mechanisms are not yet fully elucidated. There are four main pathways through which miRNAs regulate melanin production:1) miRNA may cause abnormal proliferation, differentiation, migration, and apoptosis of melanocytes, thereby affecting pigment deposition (14). For example, overexpression of miR-155 reduces the expression of melanogenesis-related genes in melanocytes and keratinocytes (184); MiR-211 inhibits UVB-induced human melanocyte migration by suppressing MMP9 (matrix metalloproteinase 9) and p53 (185). 2) miRNA targets proteins involved in melanogenesis pathways, including SOX5, β-catenin, cyclin-dependent kinase 2, and MITF (186). For example, exosomal miR-21-5p derived from peripheral blood of vitiligo patients inhibits melanogenesis in melanocytes by targeting special AT-rich sequence-binding protein-1 (SATB1) (107); MiRNA-141-3p and miRNA-200a-3p regulate melanogenesis stimulated by α-melanocyte-stimulating hormone through directly targeting MITF (187). 3) miRNA affects pigment deposition by inhibiting melanin transport.MiR-203 reduces melanosomes transport by targeting KIF5B and negatively regulating the CREB1/MITF/Rab27a pathway (188). 4) miRNA may be involved in the destruction of melanosomes. Human amniotic stem cell-derived exosomal miR-181a-5p and miR-199a inhibit melanogenesis in excessive skin pigmentation and promote melanosome degradation (131).

Exosomal miRNAs participate in melanogenesis through MAPK signaling pathways, Ras signaling pathways, Rap1 signaling pathways, cAMP signaling pathways, endocytosis, axon guidance, and Wnt signaling pathways (10). A large number of miRNAs carried by exosomes participate in intercellular communication and selectively target miRNAs in melanocytes to regulate melanin pigmentation by changing gene expression patterns and/or enzyme activity (189). Studies have shown that miR-27a-3p, miR-675, miR-145, miR-25, miR-340, miR-330-5p, miR-211, miR-155, miR-218, miR-21a-5p8, and other miRNAs affect melanogenesis through various mechanisms (175).

### 4.4 Oxidative stress-induced damage

Oxidative stress is commonly detected in vitiligo and is related to immune response and melanocyte death progression (190). The regulation of endogenous ROS production and its balance with the antioxidant system is dynamic and complex. Under physiological conditions, mild stress such as moderate sun exposure or physical exercise can induce ROS release, activating responses that maintain cellular defense barrier functions. Excessive ROS can cause the death of a small number of melanocytes by causing molecular and organelle dysfunction and exposing melanocyte-specific antigens (14, 191). Recent research has found that miRNAs regulate the response of oxidative stress in vitiligo. MiRNAs may promote the development of vitiligo by regulating the expression and function of oxidative stress-related genes in melanocytes.

Studies have shown that the expression of miR-493-3p in circulating exosomes and perilesional skin of SV patients is significantly increased. Overexpression of miR-493-3p in keratinocytes increases the concentration of dopamine in the culture supernatant, leading to a significant increase in ROS and melanocyte apoptosis, and a decrease in melanocyte proliferation and melanin synthesis in the co-culture system by targeting HNRNPU. The miR-493-3p/HNRNPU/COMT/dopamine axis may contribute to the dysregulation of melanocytes in the pathogenesis of SV (123). Oxidative stress also increases the expression of miR-25 in melanocytes and keratinocytes. Q Shi performed luciferase reporter assays, indicating that miR-25 directly regulates MITF expression by binding to the predicted target sites in the 3′UTR of its mRNAs (120). In studies of miR-25 on melanocytes, overexpression of miR-25 alone does not affect melanocyte apoptosis but can inhibit the antioxidant capacity of melanocytes by suppressing the MITF-apyrimidinic endonuclease (APE1) pathway, making melanocytes more susceptible to the effects of oxidative stress-induced apoptosis (120).

It has been reported that mesenchymal stem cell-derived exosomes can prevent H_2_O_2_-induced damage to keratinocytes, improve the antioxidant capacity of keratinocytes, and reduce the cellular response to oxidative stress (192). This provides new ideas and possibilities for the treatment of vitiligo.

## 5 Conclusions and perspective

Given the significant clinical implications of miRNAs in vitiligo, specifically targeting exosomal miRNAs as an *in vitro* strategy presents a promising approach for vitiligo treatment. In vitiligo patients, a large number of miRNAs are abnormally expressed, reflecting the activity and histological changes of the disease. These miRNAs are involved in oxidative stress responses (e.g., miR-9, miR-211), immune imbalance(e.g.,miR-21-5p,miR-133b, miR-224-3p), and melanin production (e.g.,miR-330-5p, miR-211,miR-21-5p,miR-200a-3p,hsa-miR-149-5p) (2, 163, 184). These miRNAs can reflect the activity and histological changes of vitiligo, which are considered biomarkers of vitiligo. Therefore, exosomal miRNAs become attractive diagnostic and therapeutic targets.

There are two strategies for the application of miRNAs: 1) Anti-miRNAs. Anti-miRNAs can be used to counteract the over-activation of miRNAs. For example, Short tandem target mimic-miR-143-5p can upregulate the expression of MYO5A, which in turn increases the levels of MITF, promoting melanogenesis (134). 2) miRNA Replacement. This involves reintroducing a gene-suppressor miRNA mimic or using adeno-associated virus -mediated miRNA gain-of-function to modulate gene expression. For instance,miR-211 mimic can alter the migratory capacity of melanocytes through the p53-TRPM1/miR-211-MMP9 axis, enhancing melanin levels (2, 193).

The delivery of miRNA through exosomes has attracted widespread attention. For example, Kuang et al. pointed out that in C57BL/6 mice exposed to cerebral ischemia, natural MSC-derived exosomes (rather than exosomes obtained from MSCs pre-treated with anti-miR-25-3p) regulated oligonucleotide autophagy flux and cell death by modulating the p53-BNIP3 pathway (194). Exosomal miRNAs have several excellent properties for disease treatment. Exosomes have high stability, effectively protecting miRNAs from degradation by endogenous RNases. In addition, they are widely distributed in various body fluids such as milk, urine, blood, and saliva, making them easy to isolate and non-invasively obtain. Moreover, in vitiligo, miRNAs encapsulated in exosomes have been found to exert biological functions by regulating specific aspects such as immune response, oxidative stress, and regulation of keratinocyte and melanocyte function. Importantly, many exosomal miRNAs are involved in more than two of these processes and have good consistency in their mode of action.

Currently, there are 49 clinical trials with different statuses related to miRNAs encapsulated in exosomes registered at https://clinicaltrials.gov/. Of these 49 trials, 12 are completed, 16 are active and recruiting, 5 are active but not recruiting, 2 are not yet recruiting, and 14 are unknown. It is expected that the dawn of the exosomal miRNA era will arrive.

However, there are significant challenges to be addressed when applying exosomal miRNA for disease treatment in clinical settings.

1) Exosomes have low yields, mainly obtained in limited fluids (such as culture media). Traditional exosome isolation methods, like ultracentrifugation, require multiple steps and cause significant loss and damage to exosomes, reducing their quality and purity (195). MSCs have remarkable ex vivo proliferation abilities, making them a key source for therapeutic exosome production (196). Mendt et al. developed a scalable exosome extraction method based on GMP standards, which extracts exosomes from human bone marrow-derived MSCs with yields three times higher than those from human foreskin fibroblasts (197). MSCs are widely found in adipose tissue, human umbilical cords, and bone marrow (198). Adipose tissue can be obtained from the abdominal and gluteal fat of healthy adult donors, and MSCs can be screened and cultured using specific media. Additionally, human embryonic kidney cells(HEK293) are extensively used as exosome donors in various studies due to their high transfection efficiency, ease of culture, and ability to produce large quantities of exosomes (199). Importantly, HEK293 cells can produce proteins most similar to those synthesized by the human body, with low immunogenicity, making them an ideal tool for producing protein or peptide-based drugs (200, 201).
2) In exosome-based clinical research, the practicality, safety, and compliance of exosome-related drugs are crucial issues that need to be addressed. Firstly, the choice of donor cells for exosomes is important; whether to use autologous cells or allogeneic cells with low immunogenicity requires adherence to relevant regulations and ethical review requirements. Secondly, the method of drug loading and release must be determined, optimizing the interaction between exosomes and the drug to ensure the drug’s stability and proper release rate. Thirdly, improving the targeting and tissue localization capabilities of exosomes requires developing surface modification strategies suitable for clinical application, such as using targeting peptides, antibodies, or other molecules to allow exosomes to specifically bind to target cells or tissues.

Given these limitations, future research should focus on these directions. High-yield, efficient, safe, and standardizable production of exosomes for treating vitiligo remains to be explored and developed. New effective miRNA targets for vitiligo treatment can be further investigated, with exosome delivery as a potential approach. Currently, exosome-related therapies are primarily in the preclinical stage, and their safety and efficacy need to be demonstrated. Additionally, regulatory guidelines for exosome-based drugs need to be refined. Furthermore, exosomes modified through genetic and metabolic engineering have shown more effective, stable, and safe immunomodulatory effects in the progression of autoimmune diseases (202). Researchers should continue to explore the potential value of modified exosomes in vitiligo-related therapies, focusing on aspects such as targeted delivery, circulatory stability, and biocompatibility. This will help advance the clinical application of exosomes.

In summary, our review covers the biological functions of exosomes and the involvement of exosomal miRNAs in the pathological processes of vitiligo, providing new insights for the diagnosis and treatment of the disease. Future research should delve deeper into the mechanisms between exosomal miRNAs and vitiligo progression and address the challenges of using exosomes in the clinical treatment of vitiligo.

## Author contributions

WL: Writing – original draft. YP: Writing – original draft. QH: Writing – original draft. ZS: Writing – original draft. XX: Writing – original draft. JZ: Writing – review & editing. JG: Writing – review & editing.
